# N52 monodeamidated Bcl–x_L_ shows impaired oncogenic properties *in vivo* and *in vitro*

**DOI:** 10.18632/oncotarget.7938

**Published:** 2016-03-06

**Authors:** Florian Beaumatin, Mohamad El Dhaybi, Jean-Paul Lasserre, Bénédicte Salin, Mary Pat Moyer, Mireille Verdier, Stéphen Manon, Muriel Priault

**Affiliations:** ^1^ CNRS, Institut de Biochimie et de Génétique Cellulaires, UMR5095, 33077 Bordeaux, France; ^2^ Université Bordeaux Ségalen, Institut de Biochimie et de Génétique Cellulaires, UMR5095, 33077 Bordeaux, France; ^3^ INCELL Corporation, San Antonio, TX 78249, USA; ^4^ EA 3842, Homéostasie Cellulaire et Pathologies, Université de Limoges, 87025 Limoges cedex, France

**Keywords:** Bcl-x_L_, autophagy, apoptosis, cancer, post-translational modification

## Abstract

Bcl-x_L_ is a member of the Bcl-2 family, playing a critical role in the survival of tumor cells. Here, we show that Bcl-x_L_ oncogenic function can be uncoupled from its anti-apoptotic activity when it is regulated by the post-translational deamidation of its Asn52.

Bcl-x_L_ activity can be regulated by post-translational modifications: deamidation of Asn52 and 66 into Asp residues was reported to occur exclusively in response to DNA damage, and to cripple its anti-apoptotic activity. Our work reports for the first time the spontaneous occurrence of monodeamidated Asp^52^Bcl-x_L_ in control conditions, *in vivo* and *in vitro*. In the normal and cancer cell lines tested, no less than 30% and up to 56% of Bcl-x_L_ was singly deamidated on Asn^52^. Functional analyses revealed that singly deamidated Bcl-x_L_ retains anti-apoptotic functions, and exhibits enhanced autophagic activity while harboring impaired clonogenic and tumorigenic properties compared to native Bcl-x_L_. Additionally, Asp^52^Bcl-x_L_ remains phosphorylatable, and thus is still an eligible target of anti-neoplasic agents. Altogether our results complement the existing data on Bcl-x_L_ deamidation: they challenge the common acceptance that Asn52 and Asn66 are equally eligible for deamidation, and provide a valuable improvement of our knowledge on the regulation of Bcl-x_L_oncogenic functions by deamidation.

## INTRODUCTION

Bcl-x_L_ is an oncogene whose over-expression is largely documented in cancers like colorectal adenocarcinoma [1], breast [2] and prostate cancer [3] and multiple myeloma [4]. Bcl-x_L_ was initially characterized as a Bcl-2 family member [5] displaying anti-apoptotic functions similar to Bcl-2 through its ability to prevent Bax-induced cytochrome c release from mitochondria. In spite of their 43% amino acid identity, their structural similarity, and their critical roles in cancer development and resistance to chemotherapy treatments, Bcl-2 and Bcl-x_L_ are not redundant proteins: loss of function studies in mice knocked-out for either gene allowed to discern their respective physiologic roles, Bcl-2 being required for the survival of kidney, melanocytes, stem cells and mature lymphocytes [6], and Bcl-x_L_ for neuronal and erythroid cells [7]. A comparison of the two proteins carried out in a single cellular context further highlighted (i) that Bcl-2 and Bcl-x_L_ exhibit functional differences in their inhibition of apoptosis depending on the death inducer and the pertaining signaling pathway, and (ii) that Bcl-x_L_ is more potent than Bcl-2 to warrant cell survival [8].

Another noticeable difference is the unique susceptibility of Bcl-x_L_ to undergo a post-translational modification (PTM) called deamidation in cells exposed to DNA damaging agents, [9, 10]. Deamidation is the transformation of eligible glutamine and asparagine residues into glutamate and aspartate/isoaspartate. Susceptibility for deamidation is conditioned by the primary sequence (notably if glycine is the flanking residue on the α-carboxyl side), by the lack of three-dimensional structure, and is facilitated by high temperatures, extreme pH or high ionic strength [11]. Deamidation affects a great number of proteins (human growth hormone [12], calmodulin [13], tissue plasminogen activator [14], tubulin [15], synapsin [16], Alzheimer's β-amyloid [17], histone H2B [18], protein kinase A [19], cytochrome c… [11]), and has therefore wide biological repercussions because it can lead to structural changes and/or modify their life-span.

Bcl-x_L_ was shown directly [20] or indirectly [21–23] to undergo a double deamidation of Asn^52^ and Asn^66^. Predictive algorithms calculate an equally probable deamidation for both residues [11] given their location in what is referred to as the flexible loop of Bcl-x_L_ (this region fails to adopt a defined structure according to X-ray crystallography and NMR data [24]), and the fact that they are both followed by a glycine residue. That cancer cells contain less deamidated Bcl-x_L_ than normal cells, was a first indication that deamidated Bcl-x_L_ harbors impaired anti-apoptotic functions; [20, 22, 23] *in vitro* studies further showed that Bcl-x_L_ deamidation inhibited binding to BH3-containing partners. But because deamidation produces a mixture of Asp and isoAsp, the question raised was which of the two species triggered the loss of function? Work from the Alexander lab clarified the debate opened by Deverman et al [20, 21, 25]. and showed that the prevalent occurrence of β-linked isoaspartyl residues (isoAsp^52^/isoAsp^66^), which causes more significant alterations in the structure of the protein than conversion into Asp [26], was responsible for disabling Bcl-x_L_ binding to pro-apoptotic BH3-only partners like Bim and Puma [20]. They also defined the pathway through which DNA-damage leads to NHE-1-induced intracellular alkalinization and entails Bcl-x_L_ deamidation [20]. Additionally, deamidation was also recently shown to be instrumental in the control of Bcl-x_L_ cellular amount since the deamidated protein is targeted for calpain-mediated degradation in response to cytosolic alkalinization [27]. *In fine*, Bcl-x_L_ deamidation would act both on the function and on the amount of Bcl-x_L_ to limit its pro-survival activity; as a corollary, suppression of Bcl-x_L_ deamidation would be another mechanism, unique to this member of the Bcl-2 family, implemented by tumor cells to acquire resistance to apoptosis.

Despite all these insights into deamidation-induced Bcl-x_L_ loss of function and cancer progression, three important arguments add up and highlight that the matter still deserves thriving attention: (1) cells resort to a repair enzyme called isoaspartyl methyltransferase to convert isoAsp into Asp residues, and thus eliminate the isopeptide bond which alters the conformation of proteins. Knocked out mice for such an enzyme accumulate 4–8 times more isomerized proteins than wild-type mice [28], suggesting that the restoration of isoAsp into Asp residues is potent enough to prevent the accumulation of damaged proteins and restore normal functions. More specifically regarding deamidated Bcl-x_L_, two studies showed that an isoaspartyl methyltransferase called Pimt [29] or PCMT [30] restored its anti-apoptotic functions since cells reacquired protection against stress-induced apoptosis. Therefore, the functional modifications brought by deamidation into Asp residues, and not only isoAsp should be considered. (2) Another important parameter causing a stir in the debate is that both Bcl-2 and Bcl-x_L_ are involved in the regulation of autophagy,[31, 32] a self-degradation pathway routing cellular components in vesicles called autophagosomes toward lysosomes for degradation and recycling. Autophagy is used by cells to deal with various stresses, and can be instrumental in cancer progression (reviewed in ref [33]). However, consistent with the idea that the two proteins are not interchangeable, we brought evidence of mechanistic differences in their control of autophagy, and showed that stimulation of autophagy was yet another asset allowing Bcl-x_L_ to keep death at bay [34, 35]. The functional outcome of Bcl-x_L_ deamidation on its autophagic functions has never been addressed so far. (3) Finally, to the best of our knowledge, studies investigating Bcl-x_L_ deamidation have only focused on the complete deamidation of the two Asn^52^ and Asn^66^ residues, but never on single deamidation of either residue. We therefore set out to investigate those three points. We addressed the relevance of the occurrence of single deamidation in cultured cells and *in vivo*, and report for the first time the existence of N52-monodeamidated Bcl-x_L_; we further performed an exhaustive functional characterization of this newly identified form of Bcl-x_L_ in cultured cells and *in vivo*.

## RESULTS

Monodeamidated Asp^52^Bcl-x_L_ is readily detected in control-grown HCT116 colorectal cancer cells.

**Figure 1:**
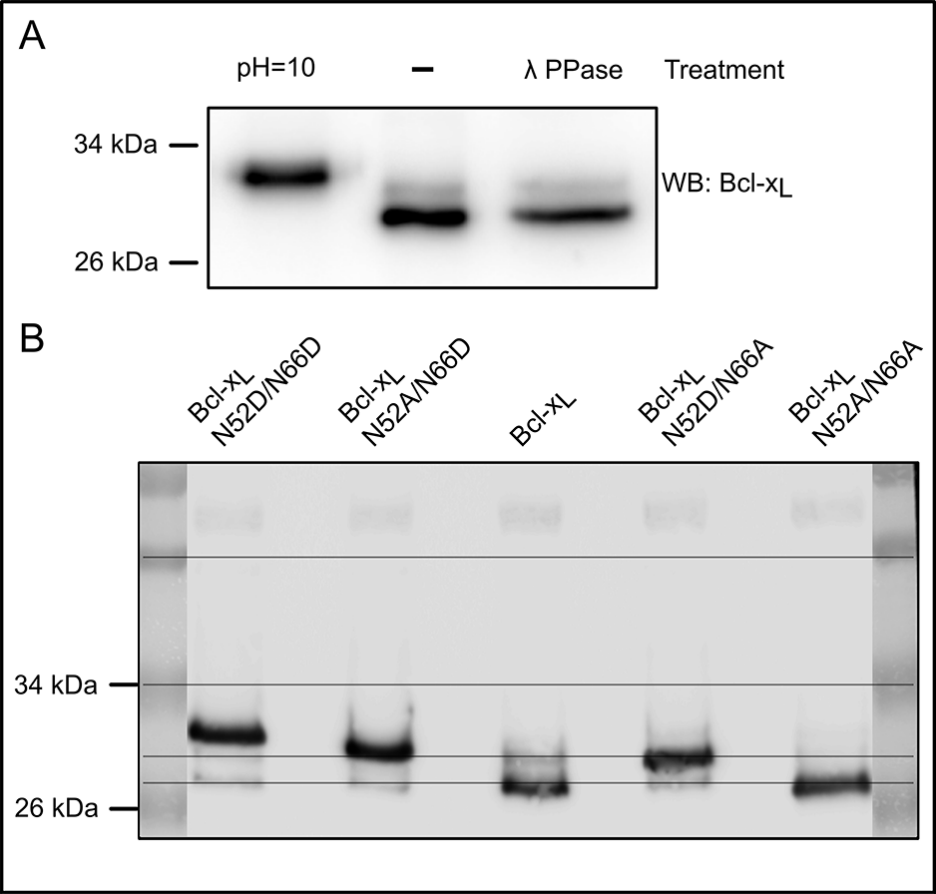
Spontaneous occurrence of monodeamidated Asp^52^Bcl-x_L_ in HCT116 colorectal cells (**A**) Total protein extracts from HCT116 cells were separated on SDS-PAGE in parallel with cell extracts submitted to alkaline treatment, or λ-phosphatase treatment. Immunodetection was performed against Bcl-x. (**B**) Total protein extracts from HCT116 cells transduced to express Bcl-xL deamidation mutants were separated on SDS-PAGE. Immunodetection was performed against Bcl-x.

A pioneering work by the Weintraub lab [21] combining SDS-PAGE of Bcl-x_L_ deamidation mutants and tandem mass spectrometry, established that deamidation can be appraised by differences in the SDS-PAGE migration profiles. Works published thereafter and reporting Bcl-x_L_ deamidation in various cell lines show western blots with the native protein at ∼30 kDa and either one [21, 22, 36] or two additional bands [20, 21, 23] migrating at shorter distances. However only the uppermost migrating band, resulting from the double deamidation of Asn^52^ and Asn^66^ of Bcl-x_L_ was functionally characterized, leaving partial deamidation of either Asn^52^ or Asn^66^ unexplored. Unlike these works, where deamidation was always induced by treatments like various DNA-damaging agents or incubation of protein extracts in an alkaline buffer, we asked whether Bcl-x_L_ could exist under partially deamidated forms in the absence of any stimulation of deamidation. In control-grown HCT116 cells, we observed that endogenous Bcl-x_L_ migrates as a doublet. The slower migrating band was not removed by λ-phosphatase treatment, and therefore did not correspond to phosphorylated Bcl-x_L_. That lambda phosphatase was active in these conditions was checked in Supplementary Figure S1. This band did not co-migrate with doubly deamidated Bcl-x_L_ contained in samples exposed to alkaline conditions (Figure 1A). We next compared the migration profiles of mutants of Bcl-x_L_ recapitulating either complete (N52D/N66D) or single deamidation (N52D/N66A and N52A/N66D) or an undeamidable form of Bcl-x_L_ (note that substitution of Asn by Asp produces a constitutively deamidated form on the position chosen, while substitution of the second Asn residue by non-deamidable Ala ensures that the protein generated will not be eligible for complete deamidation). In the stable cell lines used for this whole study, we ensured that ectopic proteins were expressed at comparable levels (Supplementary Figure S2A). The upper band in cells expressing Bcl-x_L_ unambiguously co-migrated with the singly deamidated N52D/N66A form of Bcl-x_L_ (Figure 1B), supporting that the protein undergoes the single deamidation on the residue Asn52. Those results were corroborated by two-dimensional electrophoresis coupling isoelectric focusing (IEF) experiments to SDS-PAGE, in which this modified form exhibited a more acidic pI than native Bcl-x_L_ and the non deamidable mutant N52A/N66A (as can be anticipated when Asn residues are converted into Asp), and co-migrated with deamidation mutants (Supplementary Figure S2B). From all this, we infer that Bcl-x_L_ deamidation is not always complete, and that partial deamidation of the protein exists in the absence of any DNA-damaging treatment.

### Monodeamidated Asp^52^Bcl-x_L_ is ubiquitously found in cultured cells

**Figure 2:**
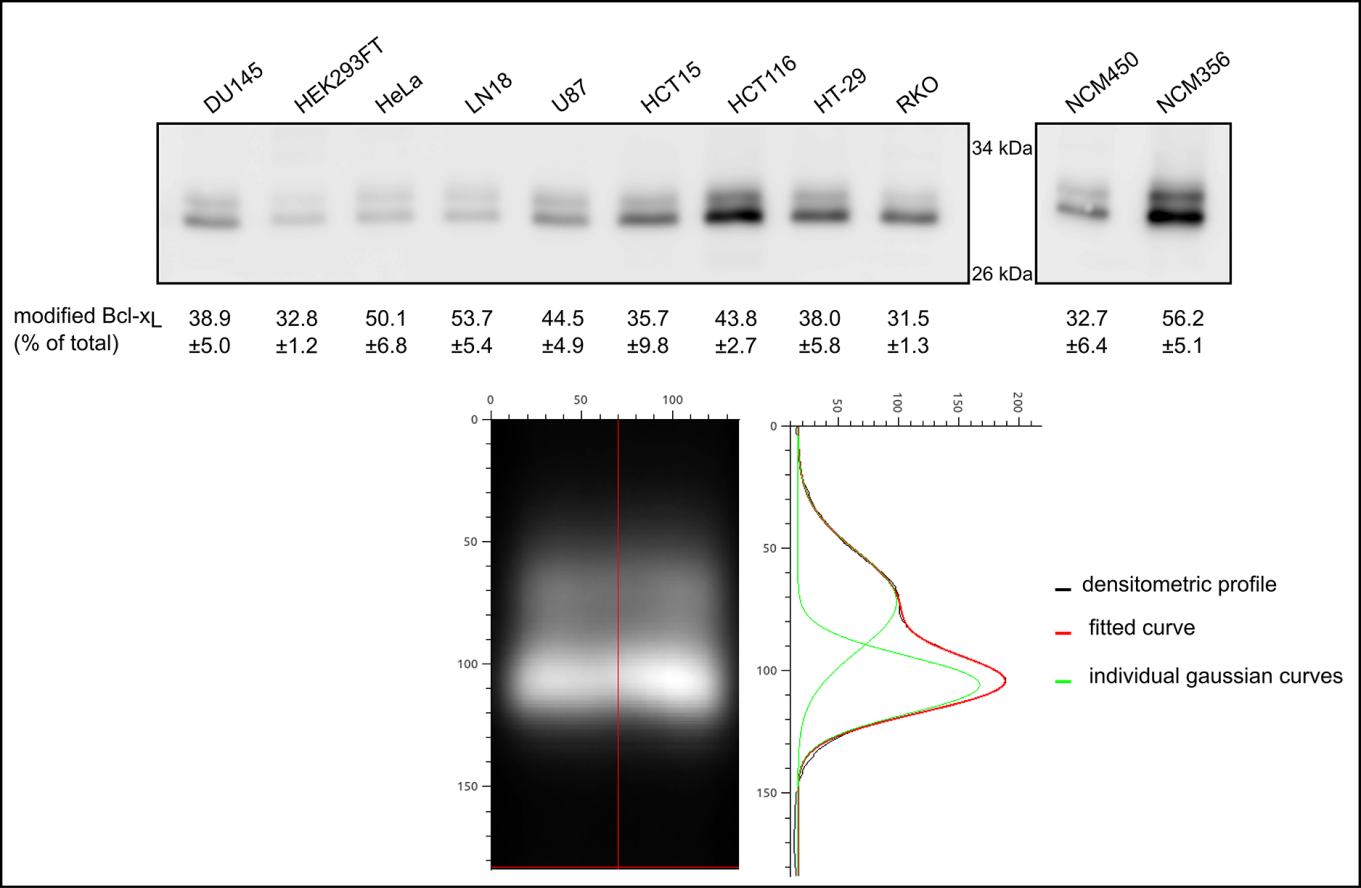
Quantification of monodeamidated Asp^52^Bcl-x_L_ in normal and cancer cell lines Total proteins were extracted from 8 cancer cell lines originating from different tissues, one transformed cell line and 2 normal cell lines derived from colon epithelium. 25 μg of proteins were separated by SDS-PAGE and an immunodetection of Bcl-x_L_ was performed. In order to resolve the signal corresponding respectively to native and to deamidated Bcl-x_L_, a Gaussian fit was applied to the densitometric profiles obtained by western blot, and the areas were measured to calculate the percentage of deamidated Bcl-x_L_ measured in each cell line. An example of the treatment is shown on HCT116 signal.

We next performed an SDS-PAGE analysis of the migration profile of Bcl-x_L_ extracted from nine different cancer cell lines and two normal cell lines, all grown under control conditions. Bcl-x_L_ unvaryingly migrated as a doublet in all the cell lines tested (Figure 2). The slower band consistently co-migrated with the singly deamidated Bcl-x_L_characterized in HCT116. An accurate quantification was performed applying a Gaussian fit on the densitometric profiles from Figure 2 to discriminate native from deamidated Bcl-x_L_. Regardless of the level of expression of Bcl-x_L_, deamidated Bcl-x_L_ accounted for no less than 31% and up to 56% of total Bcl-x_L_, which further underscores the need to address the biological relevance and the function of this singly deamidated form.

### Monodeamidated Asp^52^Bcl-x_L_ is ubiquitously found *in vivo*

**Figure 3:**
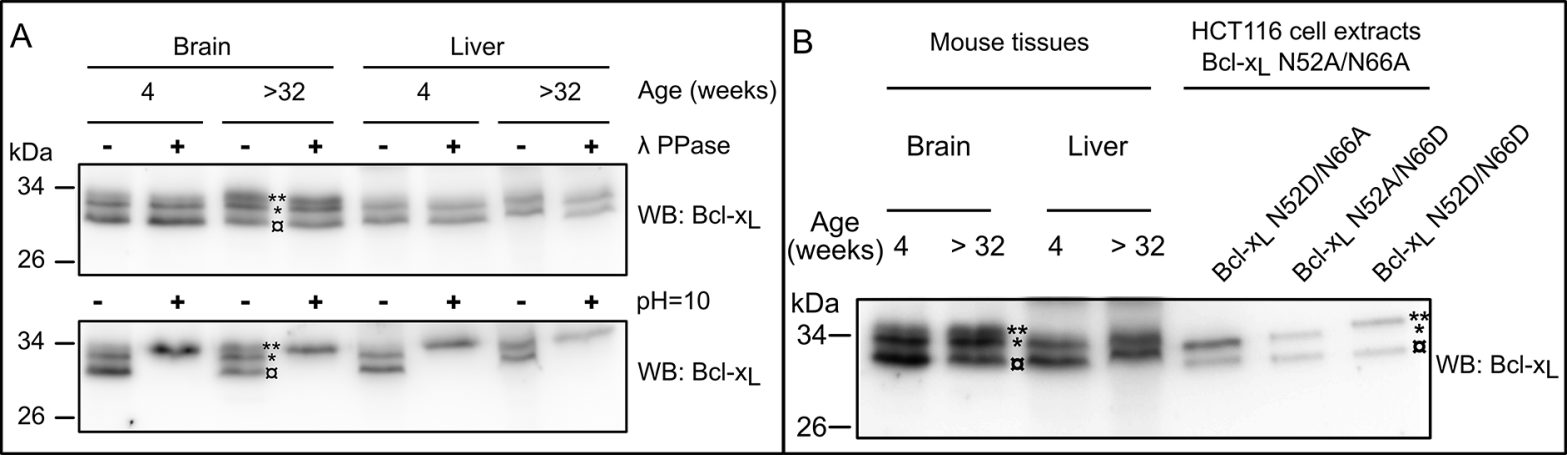
Detection of deamidated forms of Bcl-x_L_ *in vivo* (**A**) Total proteins extracts were performed from brain and liver of 4 week- or > 32 week-old mice. Samples were submitted (+) or not (−) to λ-phosphatase treatment, or submitted (+) to alkaline treatment or left at pH = 7 (−), and separated on SDS-PAGE. Immunodetection was performed against Bcl-x. The amount of proteins loaded was adjusted to obtain equivalent signals for Bcl-x_L_. Results are representative of analyses conducted with at least 3 different animals for each age. (**B**) The same tissue extracts as in panel A were loaded in parallel with total protein extracts from HCT116 cells transduced to express Bcl-x_L_ deamidation mutants. Extracts from cells expressing Bcl-x_L_ N52D/N66A, N52A/N66D and N52D/N66D were mixed with N52A/N66A to use the non deamidable form as a reference for comparisons of the migration profiles. The amount of proteins loaded was adjusted to obtain equivalent signals for Bcl-x_L_.(¤: native undeamidated/undeamidable Bcl-x_L_, *singly deamidated Bcl-x_L_; **doubly deamidated Bcl-x_L_)

We analyzed the SDS-PAGE migration profile of Bcl-x_L_ extracted from tissues of young (4 weeks) and aged mice (> 32 weeks). Extracts from leg muscles and intestine essentially showed barely detectable levels of Bcl-x_L_ regardless of the age of the mice (not shown) but Bcl-x_L_ was readily detected in brain, heart and liver tissues. It systematically migrated as a doublet or a triplet (Figure 3). The slow migrating bands (labeled *and **) were not affected upon λ-phosphatase treatment, and the highest band co-migrated with the doubly deamidated form found in alkaline treated samples. Thus, brain extracts from aged mice accumulate mono- and doubly deamidated Bcl-x_L_, while young mice present the native and monodeamidated forms. Heart extracts displayed all 3 forms of Bcl-x_L_ regardless of the age of mice (not shown). Finally, liver extracts only displayed the native and monodeamidated forms regardless of the age (Figure 3).

These results extend what we observed in cultured cells, namely that Asp^52^ momodeamidated Bcl-x_L_ is readily produced in untreated animal tissues. All the experiments above lead us to conclude that the single deamidation of Bcl-x_L_ is a widely observed process, occurring in the absence of any DNA-damaging treatment, and its abundance highlights that its functional aftermaths deserve to be thoroughly addressed.

### N52 Bcl-x_L_ deamidation does not modify the half-life of the protein

*In silico* calculation predicts equivalent deamidation rates for Asn^52^ and Asn^66^ in Bcl-x_L_ [21]. Our observation that only Asn^52^ Bcl-x_L_ is deamidated in untreated cells and tissues prompted us to question why neither Asp^66^ Bcl-x_L_ nor the double deamidated form of Bcl-x_L_ were detected. Because deamidation times the degradation of some proteins [37, 38], and because the cellular content of Bcl-x_L_ was shown to decrease in response to DNA damage [27], we hypothesized that the deamidation of Asn^66^ would shorten the half-life of Bcl-x_L_ proteins, justifying that neither Asp^66^ Bcl-x_L_ nor Asp^52^/Asp^66^ Bcl-x_L_ accumulate in untreated cells.

**Figure 4:**
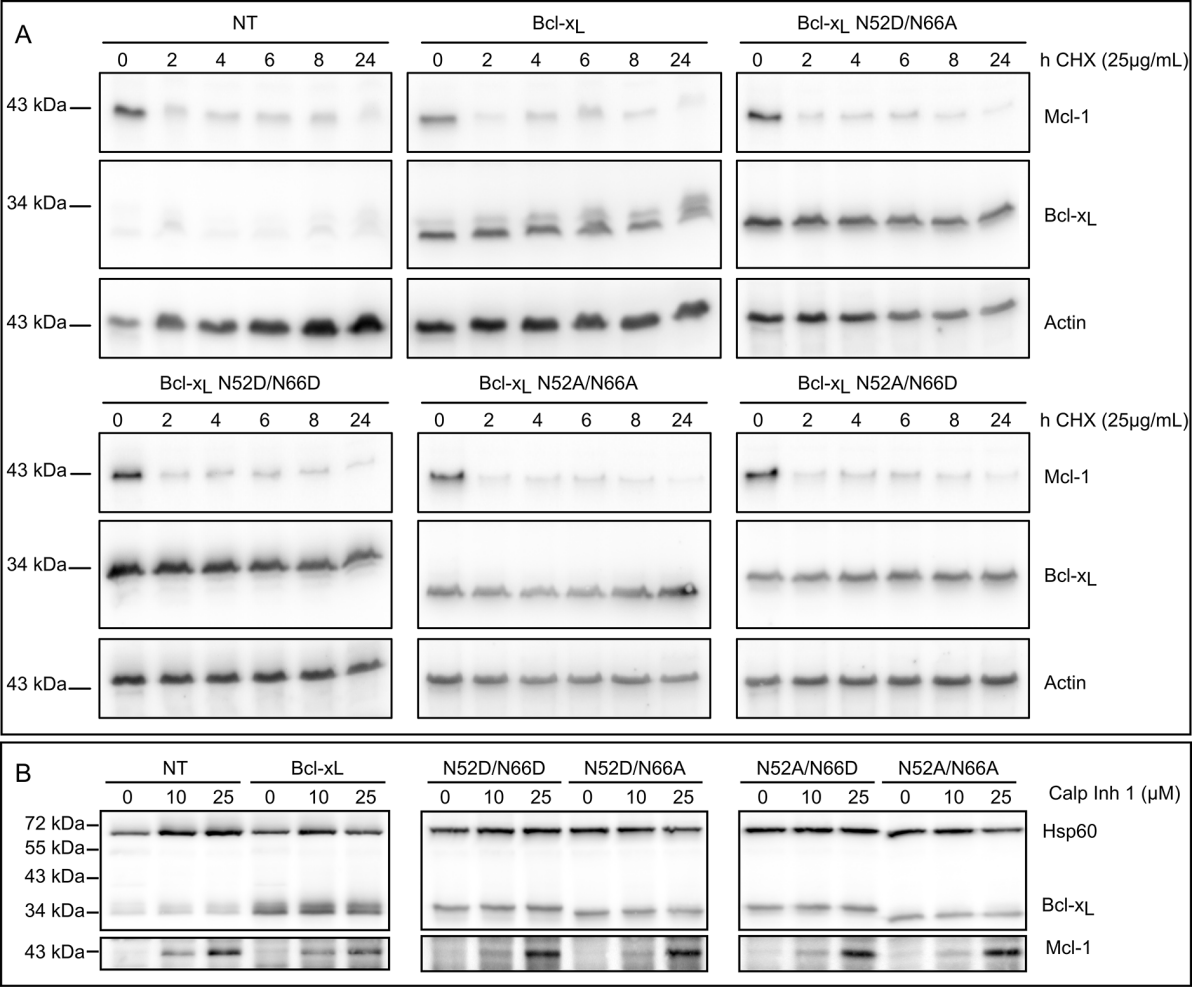
Bcl-x_L_ deamidation does not alter its half-life (**A**) Non transduced HCT116 cells (NT) and HCT116 cells transduced to express Bcl-x_L_ or the deamidation mutants were incubated with CHX for the indicated time. Total protein extracts were performed and 25 μg of proteins separated on SDS-PAGE. Immunodetection was performed against Bcl-x, actin as a loading control, and Mcl-1 as a positive control for short-lived proteins. Results are representative of 3 independent experiments. (**B**) Non transduced HCT116 cells (NT) and HCT116 cells transduced to express Bcl-x_L_ or the deamidation mutants were incubated with calpain inhibitor for 24 h. Total protein extracts were performed and 25 μg of proteins separated on SDS-PAGE. Immunodetection was performed against Bcl-x, HSP60 as a loading control, and Mcl-1 as a positive control for inhibition of calpain-mediated degradation. Results are representative of 3 independent experiments.

The degradation rate of native Bcl-x_L_ and mutants producing singly deamidated Bcl-x_L_ (N52D/N66A and N52A/N66D), double deamidated Bcl-x_L_ (N52D/N66D) and non-deamidable Bcl-x_L_ (N52A/N66A) was traced over time in the presence of cycloheximide (CHX). Mcl-1, another member of the Bcl-2 family, was used as a positive control for rapid degradation. No change in the kinetics of degradation could be observed between Bcl-x_L_ endogenous or ectopic native form, and the deamidation mutants (Figure 4A). Likewise, when we assayed whether calpain inhibition would prevent the degradation of Bcl-x_L_ deamidation mutants, as was described in cells exposed to DNA damage [27], we found no accumulation of either form of Bcl-x_L_ in control-grown cells, indicating that none of the deamidation mutants is targeted for calpain degradation (Figure 4B). From all these data, we conclude that deamidation does not alter Bcl-x_L_ half-life in cultured cells that are not challenged by DNA-damaging agents. Most interestingly, these data also provide grounding to surmise that opposite to *in silico* predictions, Asn^52^ and Asn^66^ are not equally prone to deamidation since only Asp^52^Bcl-x_L_ is observed in HCT116, and not Asp^66^ Bcl-x_L_ or Asp^52^/Asp^66^ Bcl-x_L_.

### Monodeamidated Asp^52^Bcl-x_L_ retains anti-apoptotic function

**Figure 5:**
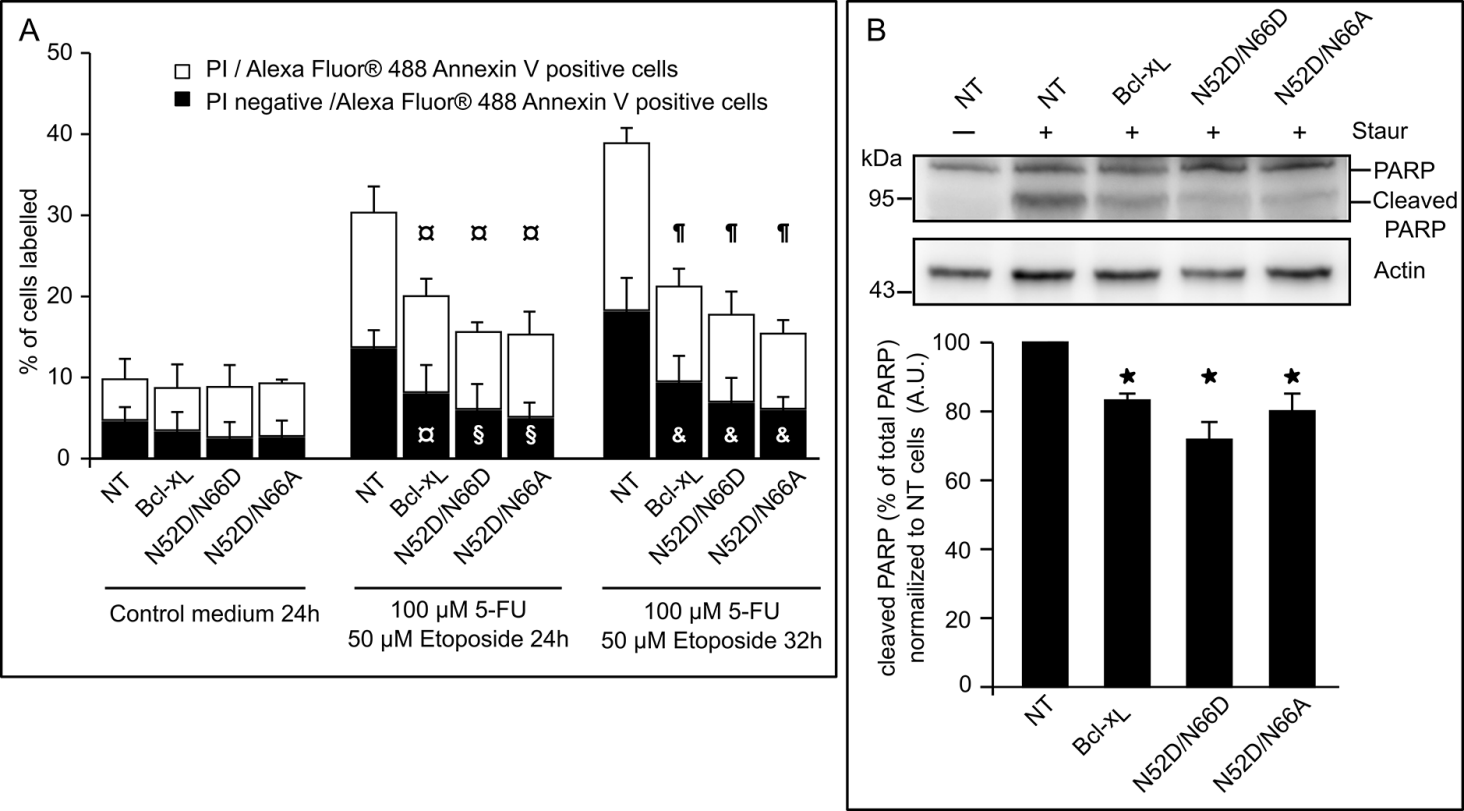
Singly and doubly deamidated forms of Bcl-x_L_ retain full anti-apoptotic activity (**A**) HCT116 cells NT or transduced to express the indicated proteins were submitted or not to DNA-damaging treatment (UV+5-FU) for the indicated times, and labeled with PI and/or Alexa Fluor^®^ 488 Annexin V. A minimum of 10 000 cells were counted, and dead cells (labeled by both dyes) or cells dying by apoptosis (labeled by Alexa Fluor^®^ 488 Annexin V) were quantified. Results are the mean of at least 3 independent experiments. Error bars show the SD. Student's test was used for statistical analysis vs NT cells. In control conditions, all *p* value are > 0,5; at 24 h of treatments, ¤ *p* < 0,1 and ^§^*p* < 0,05; at 32 h of treatments, ^¶^*p* < 0,01 and & *p* < 0,05. (**B**) (Top) Untreated NT HCT116 cells were used to show intact PARP, while HCT116 cells NT or transduced to express the indicated proteins were treated with staurosporine prior to extraction of total proteins. 50 μg of proteins were separated on SDS-PAGE, and immunodetection was performed against PARP and actin. (Bottom) Quantification of the densitometric analysis was performed on 3 independent experiments. Error bars show the SD. Student's test was used for statistical analysis vs NT cells *p < 0,05.

Bcl-x_L_ double deamidation into isoAsp^52^/isoAsp^66^, but not into Asp^52^/Asp^66^ is responsible for its loss of interaction with BH3-only partners like Bim and Puma [20]. We complemented this analysis by testing the anti-apoptotic function of the singly deamidated N52D/N66A Bcl-x_L_ in cells exposed to DNA-damage (etoposide/5-FluoroUracile (5-FU) in Figure 5A or UV-irradiation/5-FU in Supplementary Figure S3A) or treated with staurosporine (Figure 5B). Monodeamidation of N52 did neither prevent binding to Bim or Bax (Supplementary Figure S3A and S3B), nor alter its anti-apoptotic functions as assayed by FACS analysis (Figure 5A) or by PARP cleavage (Figure 5B). We also confirmed that fully deamidated Bcl-x_L_ retained anti-apoptotic functions, in keeping with the literature.

### Monodeamidation of N52 enhances Bcl-x_L_ survival autophagic functions

**Figure 6:**
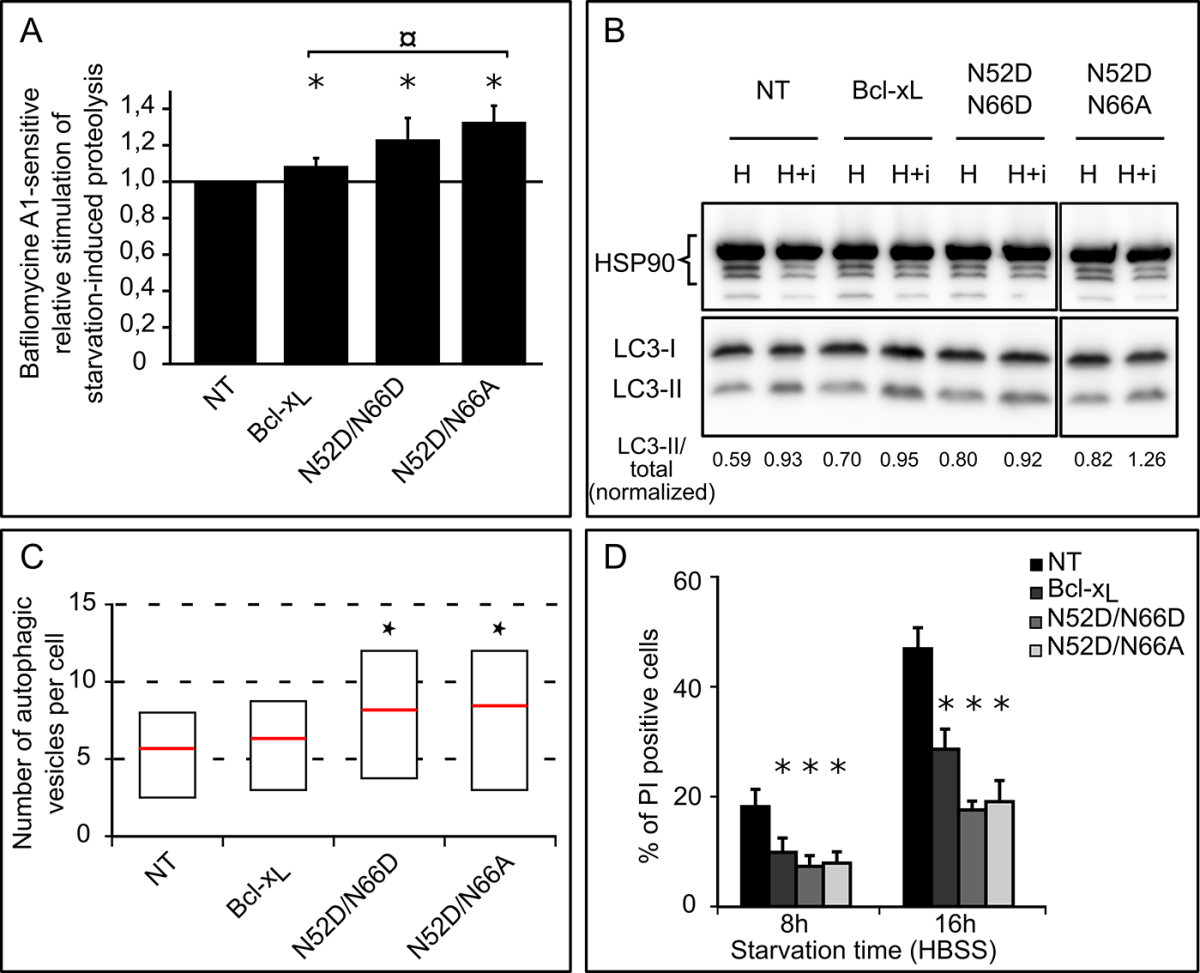
Singly and doubly deamidated forms of Bcl-x_L_ display increased autophagic activity (**A**) Relative stimulation of starvation-induced bafilomycine A1-sensitive degradation of long-lived proteins in HCT116 cells non transduced or transduced to express the indicated proteins. ^14^C-Valine-labeled cells were chased for 6 hours in HBSS or HBSS + BafA1, and proteolysis measured. Results represent the bafA1-sensitive proteolytic activities of each cell line relative to that found in NT cells. Results are the mean of 3 independent experiments. Error bars show the S.D. Normality was checked with a Shapiro-Wilk's test. One way ANOVA test followed by Tukey's test vs NT cells calculated *p < 0.05 and ¤ *p* < 0.05 vs pBcl-x_L_ cells. (**B**) Autophagic flux was measured in HEK293FT cells transiently transfected to express the indicated forms of Bcl-x_L_. Immunoblot analysis of endogenous LC3-I and LC3-II levels was performed on total proteins extracted from cells incubated in HBSS or HBSS + Baf + E64d for 60 min. HSP90 was used as a loading control to normalize the ratio LC3-II/total LC3 using the Image J software. The results are representative of 5 independent experiments. (**C**) Quantification of autophagic vesicles counted per cell on transmission electron micrographs of HCT116 cells non transduced or transduced to express Bcl-x_L_ or the deamidation mutants: cells were incubated for 6 hours in HBSS + BafA1 to measure the accumulation of autophagosomes, and samples prepared for TEM analysis. A minimum of 50 cells was analyzed for each cell line. Results are presented as box plots, with the bottom and top of the box representing respectively the first and the 3rd quartile, and the red mark indicating the mean (Student test was used to calculate *p values < 0.05 compared to NT cells). (**D**) HCT116 cells non transduced or transduced to express the indicated proteins were transferred into HBSS for the indicated time, and plasma membrane permeability was assessed by PI exclusion. A minimum of 10 000 events were quantified by FACS analysis. Results are the mean of 5 independent experiments. Error bars show the SD (Student test was used to calculate *p values < 0,002 compared to NT cells).

Ectopic expression of Bcl-x_L_ was described as of 2003 to extend survival of cells confronted to serum starvation [39], a treatment known to induce autophagy. We corroborated this finding and showed that Bcl-x_L_ exhibits pro-autophagic activity in different cell lines [34, 35]. To the best of our knowledge, the impact of Bcl-x_L_ deamidation on autophagy remains unexplored to date. We thus assessed the starvation-induced autophagic activity of HCT116 cells expressing Bcl-x_L_ N52D/N66D and N52D/N66A. The autophagic degradation of L-[^14^C]valine-labeled proteins was measured in cells starved for 6 hours (Figure 6A). Consistent with our previous findings [34], we observed that ectopic expression of Bcl-x_L_ stimulates autophagic proteolysis compared to control cells, and so did the two deamidation mutants; interestingly, Bcl-x_L_ N52D/N66A displayed a significantly greater stimulation than native Bcl-x_L_. These data were confirmed by conversion of LC3-I into its lipidated LC3-II form to measure the autophagic flux (Figure 6B), and transmission electron micrographs (Supplementary Figure S4 quantified in Figure 6C) where degradative autophagic vesicles were counted. Finally, FACS analysis of plasma membrane integrity showed that autophagy served as a cytoprotective mechanism and was not converted into a death process (Figure 6D).

### Cells expressing monodeamidated Asp^52^Bcl-x_L_ display restricted clonogenic growth and impaired tumorogenicity

**Figure 7:**
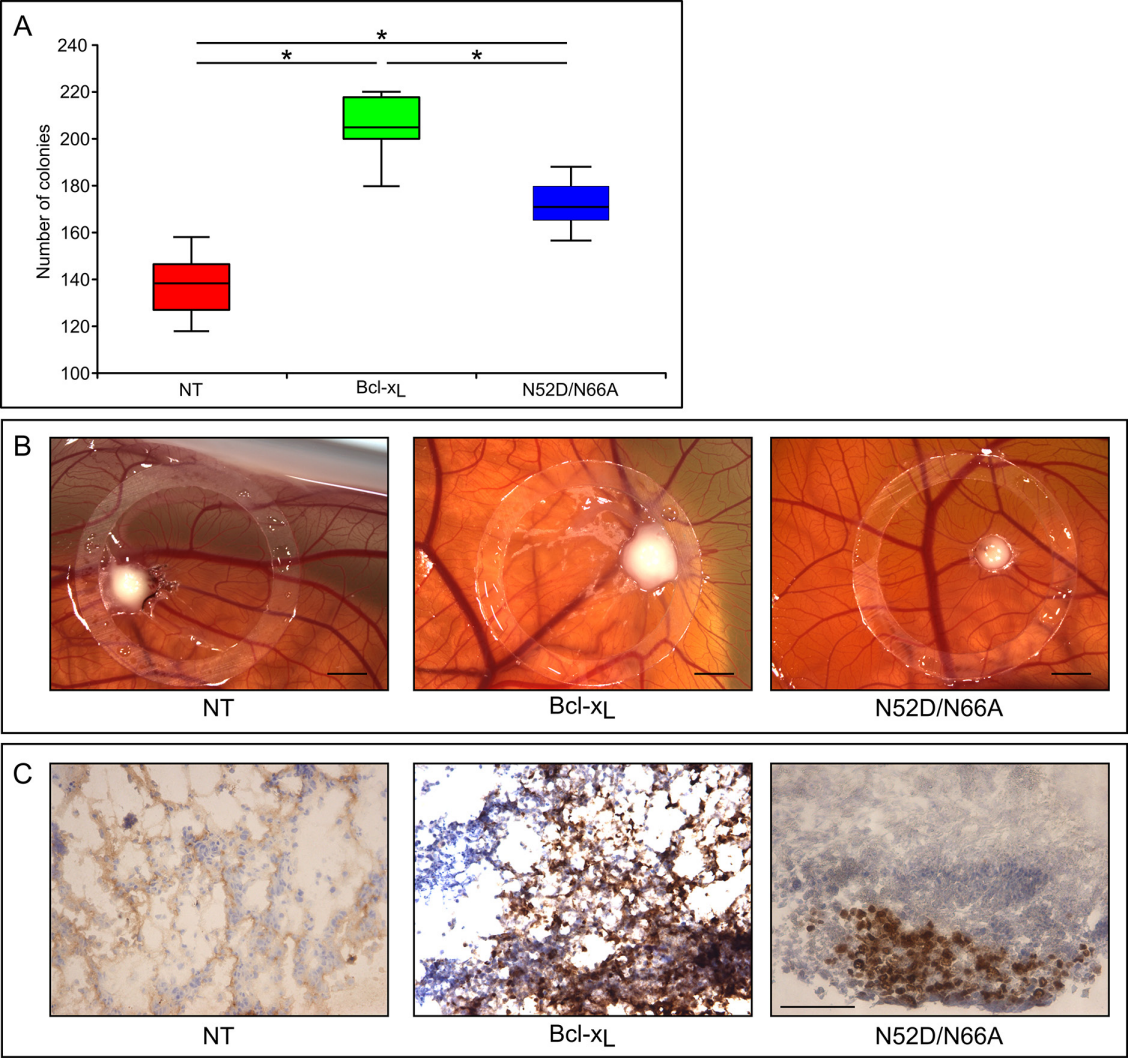
Clonogenic and tumorogenic potential of the singly deamidated form of Bcl-x_L_ (A) HCT116 cells non transduced or transduced to express the indicated proteins were plated in semi-polymerized collagen medium (500 cells per well in 6-well plates) The colonies were stained and counted 21 days later. Results are the mean of 3 independent experiments. Normality was checked with a Shapiro-Wilk's test. One way ANOVA test followed by PLSD Fisher test was used for statistical analysis (*p values < 0,0001); error bars show the standard error. (**B**) Macroscopic features of the chick chorioallantoic membranes at day 3 after implantation of the indicated HCT116 cells. Scale bar = 0, 2 cm. (**C**) Sections of the tumors originating from the indicated HCT116 cells were labeled with antibodies directed against Ki67 proliferation marker. Scale bar is 50 μM.

We next assayed the clonogenic and tumorigenic potential of singly deamidated Bcl-x_L_. Anchorage-independent growth of cells in a semi-polymerized medium showed that the number of colonies formed by HCT116-Asp^52^Bcl-x_L_ was significantly lower than HCT116-Bcl-x_L_ (Figure 7A). Hence, monodeamidation of Bcl-x_L_ on Asn^52^ restricts the clonogenic properties of Bcl-x_L_ *in vitro*.

We finally used chick embryo chorioallantoic membrane (CAM) as an *in vivo* model to study tumor formation upon implantation of HCT116 cells. Three days after inoculation, tumors xenografts were visible and were excised from the CAMs. Tumors implanted with HCT116-Bcl-x_L_ were conspicuously bigger than those derived from non transduced cells, validating CAM as a relevant model to assay Bcl-x_L_ tumorogenic potential. Interestingly, tumors derived from HCT116-Asp^52^Bcl-x_L_ were smaller than those derived from HCT116-Bcl-x_L_ (Figure 7B), indicating that monodeamidation impairs Bcl-x_L_ tumorogenic properties.

This result was substantiated by immunohistochemistry analyses of the mitotic index with Ki67 labeling (Figure 7C). More than 90% of the tumor cells derived from HCT116-Bcl-x_L_ were Ki67 positive, but the labeling was dramatically decreased in those originating from cells expressing monodeamidated Asp^52^Bcl-x_L_, indicating a much lower proliferation index.

Altogether, both *in vitro* and *in vivo* tests showed that monodeamidation of Asn^52^ impairs Bcl-x_L_ clonogenic and tumorigenic properties.

### Monodeamidated Asp^52^Bcl-x_L_ remains a target for a selective class of anti-neoplasic agents

**Figure 8:**
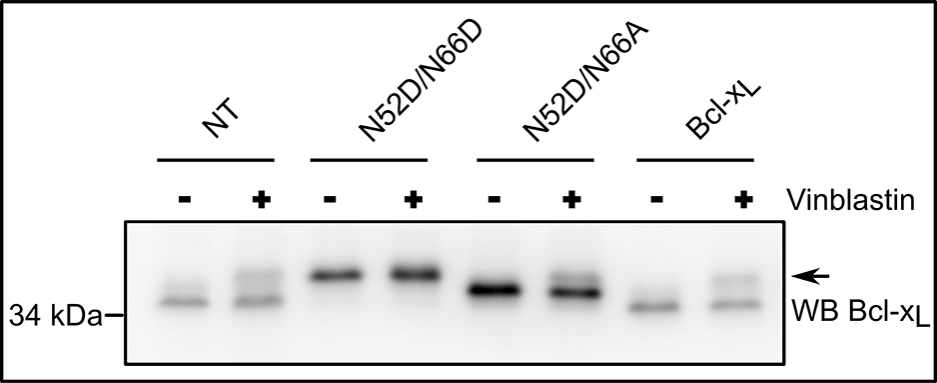
Monodeamidated Asp^52^Bcl-x_L_ can be phosphorylated in response to anti-neoplasic agents HCT116 cells non transduced or transduced to express the indicated proteins were treated or not with 100 nM vinblastin for 24 h, a treatment known to destabilize micro-tubules and induce Bcl-x_L_ phosphorylation (arrow). Total proteins were extracted and separated on SDS-PAGE. The amount of proteins loaded was adjusted (especially for NT cells) to obtain detectable signals for Bcl-x_L_, after immunodetection with anti-Bcl-x antibody. Results are representative of 5 independent experiments.

We demonstrated so far that monodeamidated Asp^52^Bcl-x_L_ has decreased clonogenic and tumorigenic activities, but retains unscathed anti-apoptotic functions. Therefore we last asked whether this modified form would still be an eligible target for anti-neoplasic treatments that prove toxic for cancer cells because they trigger Bcl-2/Bcl-x_L_phosphorylation and hence abrogate their anti-apoptotic activity. Taxol and vinblastin are such compounds, that alter the architecture of microtubules and initiate pathways leading to Bcl-2/Bcl-x_L_ phosphorylation [10]. In HCT116 cells, vinblastin treatment triggered the phosphorylation of native Bcl-x_L_ (endogenous or ectopic) and also of the singly deamidated mutant Bcl-x_L_ N52D/N66A (Figure 8). Therefore we conclude that monodeamidated Asp^52^Bcl-x_L_ is still susceptible to phosphorylation in response to microtubule-targeting anti-neoplasic agents. Such a result fosters the idea that the concerted use of drugs can be envisaged to target the anti-apoptotic activity of singly deamidated Asp^52^Bcl-x_L_and further limit its oncogenic properties.

## DISCUSSION

A growing body of evidence supports the fact that Bcl-2 family members are not only key regulators of cell apoptosis, but also actively participate in the regulation of vital cellular functions. As a consequence, restricting the oncogenic properties of the anti-apoptotic proteins of this family to their ability to oppose apoptosis is now dated. The work presented here brings further support to this concept, as we identified a novel PTM-modified form of Bcl-x_L_ which retains full anti-apoptotic functions, but shows impaired oncogenic properties.

### Monodeamidated Asp^52^Bcl-x_L_ is ubiquitously found

The confrontation of several articles dealing with the PTM of Bcl-x_L_ led us to elaborate on the occurrence of monodeamidated Bcl-x_L_. To the best of our knowledge, articles reporting on Bcl-x_L_ deamidation only envisage this modification in the context of DNA damage, and led to the characterization of the doubly deamidated protein on Asn^52^ and Asn^66^. We found that in the absence of any genotoxic cue, a monodeamidated form of Bcl-x_L_ on Asn^52^ was readily observed in various types of normal and cancer cultured cells (Figures 1 and 2), and all the mouse tissues where Bcl-x_L_ was detectable (Figure 3).

### Deamidation of Asn^52^ as a telltale of structural reorganization

The detection of Asp^52^Bcl-x_L_ highlighted that Asn^66^ seemed refractory to deamidation; this raised the salient point that opposite to *in silico* predictions, Asn^52^ and Asn^66^ are not equally prone to deamidation in cells. One plausible hypothesis is that the region described by X-ray crystallography and NMR data (on the isolated, purified protein) as a large unstructured loop containing Asn^52^ and Asn^66^, could well adopt a defined structure in cells, due to the protein insertion into membranes, or to its interaction with a binding partner, or both. As a result, deamidation of Asn^66^ could become improbable while leaving Asn^52^ unaffected. To our knowledge, this is the first time that a set of experiments portends the structuring of Bcl-x_L_ large loop, and could designate this region as a site of intervention for drug design to abolish its oncogenic functions.

### Deamidation and Bcl-x_L_ half-life

**Figure 9:**
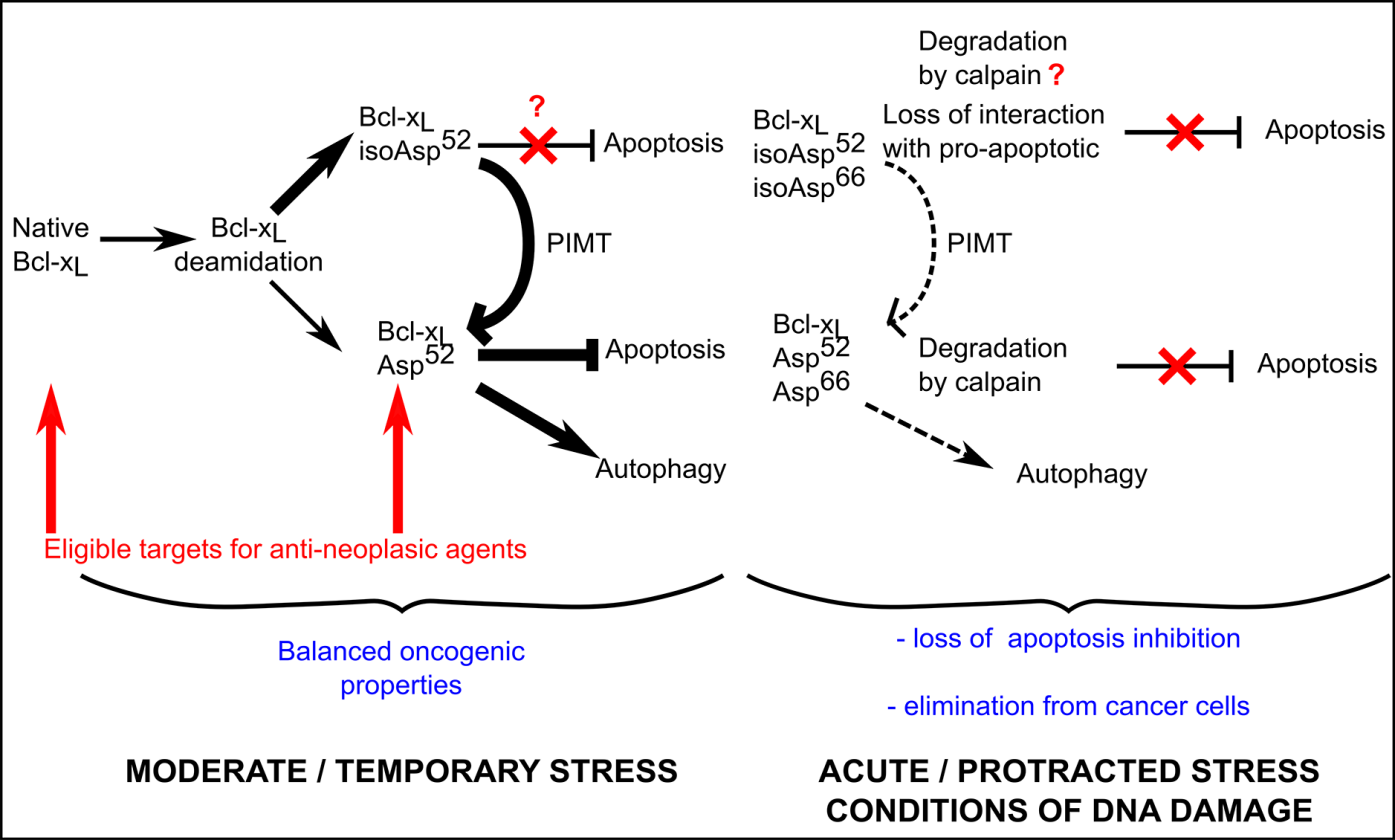
Proposed contribution of Bcl-x_L_ deamidation to cell survival/death balance Cells grown under normal conditions (confronted to a moderate stress corresponding to steady state metabolism) produce both native and monodeamidated Bcl-x_L_. The literature indicates that deamidation produces 70–85% isoAsp residues [15] which are then very efficiently converted into Asp residues by the repair enzyme PIMT/PCMT. Whether the structural alteration of isoAsp^52^Bcl-x_L_ entails functional alterations is currently unknown. We described here that Asp^52^Bcl-x_L_ provides efficient protection against apoptosis and stimulated autophagic activity compared to the native protein *in vitro*, while displaying restricted clonogenic and tumorigenic properties *in vivo*. As cells constantly express a mixture of native and Asp^52^ monodeamidated Bcl-x_L_, it appears that they deal with a net outcome of balanced oncogenic species. When cells are exposed to protracted/acute stress, the complete deamidation of Bcl-x_L_ is stimulated, leading to the preponderant formation of isoAsp^52^/isoAsp^66^Bcl-x_L_. Because deamidation targets many proteins, repair enzymes are overwhelmed and the restoration of active conformation of deamidated proteins is less efficient. As a result, cells deal with species of Bcl-x_L_ that have either lost their ability to provide protection against apoptosis (isoAsp^52^/isoAsp^66^Bcl-x_L_) and/or are targeted for degradation by calpain (whether calpain targets the isoAsp or the Asp forms of doubly deamidated Bcl-x_L_ was not investigated [27]); as a result, cells can no longer resort to Bcl-x_L_ anti-apoptotic functions, and either trigger cell death or engage on the muti-step process leading to cellular transformation. Cells that are prone to undertake the later modifications are reported to suppress Bcl-x_L_ double deamidation in order to still resort to the oncogenic properties of the native protein.

The literature shows that cells resort to Bcl-x_L_ double deamidation to regulate both its function and cellular amount under conditions of DNA damage (Figure 9). In keeping with this observation, the fact that Asp^66^Bcl-x_L_ was never detected could have been a consequence of an extremely rapid degradation. We refuted this hypothesis when we analyzed the half-life of the deamidation mutants (Figure 4A). These experiments revealed instead that in control-grown cells, all the deamidation mutants proved extremely stable. Further, the cellular amount of all the deamidation mutants remained unchanged when calpain was inhibited (Figure 4B). Therefore, our work nicely complements the data reported by Dho et al. and show that the regulation of Bcl-x_L_ observed under acute stress does not apply to control-grown cells. Deamidation (either single or double) is not a sufficient cue to trigger calpain-mediated degradation; instead the process seems to require the peculiar cellular context of DNA damage and the ensuing cytoplasmic alkalinization [27].

### Drawing the inventory of monodeamidated Asp^52^Bcl-x_L_ functions

We found that monodeamidated Asp^52^Bcl-x_L_ opposes apoptosis with the same efficiency as native Bcl-x_L_ or the double deamidated N52D/N66D Bcl-x_L_, the latter observation corroborating others’ works [20, 21, 25]. Indeed, both the protection against apoptosis induced by metabolic inhibitors or DNA-damaging agents and the interaction with pro-apoptotic partners like Bim and Bax were unchanged (Figure 5, Supplementary Figure S3). However Asp^52^Bcl-x_L_ gains increased autophagic activity compared to native Bcl-x_L_, and as a result improves the ability of cells to endure nutrient/serum starvation (Figure 6).

Consequently, although the literature on deamidation casts special emphasis on the conversion of Asn into isoAsp residues due to the structural modification entailed, our results show that deamidation into Asp residues can generate gain of function proteins. The legitimate speculation stemming from this is that cells exposed to moderate stress (thus generating singly deamidated Bcl-x_L_), and containing an active repair enzyme PCMT/PIMT that converts isoAsp into Asp residues might not only be more efficiently protected against apoptosis because they restore the structure of Asp^52^Bcl-x_L_, but may also be more prone, as they produce Asp^52^Bcl-x_L_, to induce cytoprotective autophagy as a mechanism opposing the stresses accompanying cellular transformation.

### Monodeamidation and anti-cancer treatments

That monodeamidated Asp^52^Bcl-x_L_ was ubiquitously found and accounts for such high proportions compared to native Bcl-x_L_ (Figure 2) proves that it can no longer be legitimately ignored. Our finding that monodeamidation of Asn52 acted as an intra-molecular safeguard that limits Bcl-x_L_ oncogenic properties (Figure 7) and that it could still be phosphorylated in response to microtubule-targeting anti-neoplasic agents (Figure 8) leads us to elaborate the following rationale: in the early stages of tumorigenesis, intracellular stresses, although intense, do not qualify to trigger Bcl-x_L_ deamidation; hence the stimulation of monodeamidation in combination with microtubule-targeting anti-neoplasic agents could prove an interesting point of intervention to efficiently abrogate Bcl-x_L_ contribution to cellular transformation (Figure 9). Future work in this direction still awaits the avenue of chemical compounds driving the selective deamidation of Bcl-x_L_, or pending that, the identification of specific binding partners and private pathways controlled by monodeamidated Bcl-x_L_.

## CONCLUSIONS

In light of the conservation of the deamidation-prone asparagines in Bcl-x_L_ throughout evolution [27], this PTM clearly stands as a multifaceted process modifying proteins structure and sequence: in concert with repair enzymes, deamidation acts as a fine tuning system partitioning Bcl-x_L_ proteins in two sub-populations harboring different functions and depending on different regulation pathways (Figure 9).

Shortly after its discovery 20 years ago^5^ Bcl-x_L_ was identified as one powerful obstacle to successful chemotherapy [40] because it acts at multiple steps of the process of drug resistance acquisition. Our findings that (1) Bcl-x_L_ large loop is likely to adopt a defined structure in cells, (2) that monodeamidation acts as an internal safeguard for Bcl-x_L_oncogenic properties, and (3) that Asp^52^Bcl-x_L_ remains a target for anti-neoplasic agents inducing its phosphorylation, are of particular importance for therapies designed to limit the survival means cancer cells can resort to. Bcl-x_L_ could prove a valuable key to provide at once several points of intervention to kill malignant cells. Our work contributes to characterize such a multi-modal action, and indicates the future directions to enable the identification of new compounds targeting Bcl-x_L_.

## MATERIALS AND METHODS

All cell culture material was from Invitrogen. All chemicals were from Sigma Aldrich. Vinblastin was from MP Biomedical. Staurosporine (S-9300) and Bafilomycin A1 (B-1080) were from LC Laboratories.

### Cell lines and cultures

HCT116 cells were obtained from Dr Vogelstein (Baltimore, USA) and grown in Opti-MEM^®^ supplemented with 5% fetal calf serum (FCS). HEK293FT cells were grown in DMEM containing 4, 5 g/L glucose supplemented 10% FCS. Growth media contained penicillin (100 U/mL) and streptomycin (100 μg/mL). NCM460 and NCM356 cells [41] were received through a Material Transfer Agreement with INCELL Corporation, San Antonio, Texas, USA. These cells were grown in M3Base medium (INCELL) supplemented with 10% FCS.

Deamidation mutants of Bcl-x_L_ were generated by site-directed mutagenesis: N52A: 5′- GAGACCCCCAGTGCCATCGCCGGCAACCCATCCTG-3′; N66A: 5′- CAGCCCCGCGGTGGCCGGAGCCACTGGCC-3′; N52D: 5′- CCCAGTGCCATCGATGGCAACCCATCCT G-3′; N66D: 5′- CAGCCCCGCGGTGGATGGAGCCA CTG-3′.

The template used for the mutagenesis was a plasmid containing a cDNA of Bcl-x_L_ resistant to short hairpin RNA (shRNA) interference owing to four silent point mutations in the region targeted by Bcl-x_L_ shRNA (5′- AGG AUA CAG CUG GAG UCA G -3′).

Recombinant lentiviruses were engineered, produced, and titrated as previously described [34]. A multiplicity of infection of 4 was used to generate stable cell lines from HCT116 cells.

### Animals

Wild type BALB/c (BALB/c WT) were purchased from Charles Rivers Laboratories and euthanized at University Bordeaux animal facilities in strict accordance with European legal and ethical rules. Organs were collected, and the powder resulting from cryogenic grinding was solubilized in RIPA buffer (100 mM Tris, 0,5% NP-40, 0,5% sodium-deoxycholate, 0,1% SDS supplemented with proteases inhibitor Mini^®^ (Roche Diagnostics)).

### Western blot

Total proteins were extracted in RIPA buffer, were separated on SDS-polyacrylamide gel electrophoresis (PAGE), transferred onto polyvinylidene fluoride (PVDF) membrane (Millipore) and western-blots revealed with ECL (Perkin Elmer). Antibodies used are: rabbit anti-LC3 (#L7543, Sigma Aldrich), rabbit Anti-Bcl-x (#610213, BD Transduction Laboratories), rabbit anti-Mcl-1 (S-19), goat anti-HSP60 (K-19) and mouse anti-HSP90 (sc-69703) (Santa Cruz Biotechnology), mouse anti-actin (#MAB1501R, Millipore) and rabbit anti-PARP (#11835238001, Roche Diagnostics). Horseradish peroxidase-conjugated secondary antibodies were from Jackson Immunoresearch. Densitometric profiles were analyzed either with ImageJ when the peaks were separate, or with QtiPlot software when the peaks were overlapping.

### *λ*-Phosphatase treatment

Total proteins were extracted in the presence of phosphatase inhibitors (10 mM NaF, 1 mM Na_3_VO_4_, 1 mM phenylmethanesulfonylfluoride). The extracts were then diluted 10 times in the dephosphorylation buffer. Dephosphorylation used 200 units of λ-phosphatase (#P0753S, Biolabs) for 2 h at 37°C. The reaction was stopped by 4% SDS, 125 mM Tris pH = 6.8, 20% glycerol, 0.002% (w/v) bromophenol blue.

### *In vitro* deamidation reaction

Total protein extracts were incubated with 25 mM glycine-NaOH, pH = 10 for 24 h at 37°C.

### Fluorescence-activated cell-sorting analysis

Apoptosis was induced by 100 μM 5-FU and 50 μM etoposide for 24 and 32 h. Cells were labeled with propidium iodide (PI) and/or Alexa Fluor^®^ 488 Annexin V according to the manufacturer's instructions (Dead Cell Apoptosis Kit, Invitrogen). Proper compensation values were applied prior to quantification. For survival under autophagic conditions, cells were transferred into HBSS for 8 h and 16 h, harvested and labeled with PI. Quantifications were made using Accuri flow cytometer and the C-flow software.

### PARP detection

Cells were treated for 8 h with 50 μM staurosporine. Immunodetection of intact and cleaved PARP was performed after protein extracts were separated on 10% SDS-PAGE.

### Autophagic proteolysis assay

The degradation of radioactive L-[^14^C]valine-labeled proteins was measured as previously described [34]. Briefly, cells were incubated for 24 hours in complete medium with 0.1 μCi L-[^14^C]valine to label total proteins. Radioactivity was further pre-chased for 1 hour in complete medium in the presence of an excess of L-valine (10 mM) to remove the contribution of short-lived protein degradation. Finally, cells were incubated for 6 hours either in complete medium or in Hank's Buffered Salts Solution (HBSS) (autophagy was induced by amino acids and serum starvation in Hank's Buffered Salts Solution buffered with 2.2 g/L NaHCO_3_) in the presence or in the absence of the lysosomal inhibitor Bafilomycin-A1 and with an excess of L-valine. Supernatants were collected and free amino acids precipitated with 80% trichloroacetic acid (TCA), while proteins in adherent cells were precipitated with 10% TCA. Radioactivity was quantified in a scintillation liquid analyser Tri-carb 2100TR (Packard). Proteolysis is expressed as the percentage of free radio-activity released in the supernatant relative to the total radioactivity.

### Autophagic flux assay

HEK293FT cells were transfected with a calcium phosphate method. 48 h later, they were incubated under control conditions or washed and transferred for 60 minutes into starvation medium (HBSS) or starvation medium supplemented with the lysosomal ATPase inhibitor Bafilomycin A1 (0.1 μM) and the cysteine protease inhibitor E64d (10 μg/mL) which inhibits cathepsins B, H and L. Total proteins were immediately extracted and separated by SDS-PAGE.

### Protein half-life determination

Sub-confluent cells were treated with CHX (25 μg/mL) for 0–24 h to inhibit cytosolic protein synthesis. Cell lysates were then separated on 12% SDS-PAGE.

### Anchorage-independent growth assay

HCT116 cells were resuspended in OptiMEM medium and mixed with collagene type I extracted from 10-week old rat tails; 500 cells were plated in triplicate in 6-well plates, and fresh OptiMEM was layered over the semi-polymerized medium. The medium was refreshed every 3 days, and 21 days after plating, colonies were fixed with paraformaldehyde, satined with crystal violet and counted. Normality was assayed with a Shapiro test, and statistical difference was assayed by a Fisher test.

### *In vivo* tumorogenic assays in chick chorioallantoic membranes

Embryonated eggs were purchased from a local hatchery (HAAS, Kaltenhouse) and incubated 2, 5 days in a 65% humidified chamber at 38°C, before opening. The incubation of chick chorioallantoic membranes was prolonged for 1 more week. At day 9, three million cells were deposited after gentle scratching of the CAM surface, into a 1 cm Ø Teflon ring. Three days after inoculation, tumor were excised, imaged under a MZFL3 stereomicroscope (Leica), and submitted to immunohistochemistry analyses.

### Histological analyses

Tumors were immediately frozen and embedded in OCT compound. They were left at −80°C until sectioning. 7 μm-thick tumor sections were cut with a cryo-microtome (Leica CM 3050). Samples were fixed in 4% paraformaldehyde, and saturated in 1% BSA before incubation with Ki67 antibody (M7240, clone MIB-1, Dako). Detection was performed with the EnVision System-HRP-DAB (K4010) from Dako, according to the manufacturer's instructions. Finally, sections were counter-stained with hematoxylin (Merck) before mounting on slides with the Coverquick mounting medium (Labonord). The observation was done under the Leica DM-RX microscope.

## SUPPLEMENTARY MATERIALS FIGURES



## Abbreviations

PTM: post-translational modifications
CHX: cycloheximide
CAM: chicken chorioallantoic membrane.
