# Supplementary material for: N52 monodeamidated Bcl–xL shows impaired oncogenic properties in vivo and in vitro
Source: Oncotarget. 2016 Mar 6;7(13):17129–43. doi: 10.18632/oncotarget.7938 (PMC4941376; doi:10.18632/oncotarget.7938)
Supplement: Supplementary file 1 [file oncotarget-07-17129-s001.pdf]

N52 monodeamidated Bcl-x_L shows impaired oncogenic properties *in vivo* and *in vitro*

Supplementary Materials

Two-dimensional electrophoresis (IEF/SDS-PAGE)

HCT116 cells transduced to express Bcl-x_L or the deamidation mutants were grown in control medium and the expression of endogenous Bcl-x_L silenced by infection with lentiviral particles delivering Bcl-x_L shRNA. Total proteins were extracted and the lysis buffer was replaced by IEF buffer (7 M urea, 2 M thio-urea, 4% Chaps w/v, 30 mM Tris) by centrifugation. Protein concentration was determined by Bradford assay. Non-linear Ready Strip IPG strips (pH 3–10 NL, 11 cm) (Bio-Rad) were rehydrated over-night at 50 volts with rehydration buffer (7 M urea, 2 M thio-urea, 1% w/v CHAPS, 0.4% w/v DTT, 0.5% v/v IPG (immobilized pH gradient) buffers, 0.002% v/v bromophenol blue) and 50 µg of total proteins. IEF was then carried out on Protean IEF cell (Bio-Rad) with the following settings: 60 V for 2 h, gradient step of 60–500 V for 2 h, 500 V for 2 h, gradient step of 500–1000 V for 2 h, 1000 V for 2 h, gradient step 1000–3000 V for 5 h and a constant step at 3000 V for 6 h at 20°C with a maximum current setting of 50 µA/strip in an Protean IEF unit. Individual strip were equilibrated for 5 min in an equilibrating buffer containing 1% SDS (w/v), 125 mM Tris, pH = 6.8 and then dipped into equilibrating buffer supplemented with 50 mM dithiothreitol (Sigma Aldrich) for 15 min. Individual lanes were subsequently soaked in equilibrating buffer supplemented with 125 mM iodoacetamide (Sigma Aldrich) for 15 min. An ultimate washing step of 5 min was performed in the equilibrating buffer without supplement. The IPG strips were then sealed with 0.5% agarose in SDS running buffer at the top of 12% SDS-PAGE gel (200 × 160 × 1 mm). After migration at 200 V during 4 h, the proteins are transferred onto PVDF membrane. Western blots against actin and Hsp60 (Santa Cruz Biotechnology) proteins were used as standards to position the migration profiles of Bcl-x_L and of the deamidation mutants.

Bcl-x_L /Bim co-immunoprecipitation assays

HCT116 cells transduced to express Bcl-x_L or the deamidation mutants were used for the co-immunoprecipitation assay with Bim. Cells were left untreated or treated with UV (45 sec, 450 µJ) + 5-FU (40 µM, 16 h). They were then collected and were lysed in 1 × IP buffer (Sigma Aldrich). Co-immunoprecipitations were done overnight with 1 mg of protein extracts and 2 µg of anti-Bim antibody (Rabbit anti-Bim, Sigma Aldrich). 50 µL of Protein G-agarose beads were added and incubated for 6 hours at 4°C. IPs were washed with 7 × 500 µL of 1 × IP buffer, and proteins were eluted with 30 µL of dissociation buffer (2% SDS, 125 mM Tris pH = 8.8, 20% glycerol, 0.002% (w/v) bromophenol blue).

Half of eluted fractions were separated on 12% SDS-PAGE prior to evaluation by western blots of the amount of immunoprecipitated Bim and co-immunoprecipitated Bcl-x_L.

Bcl-x_L /Bax co-immunoprecipitation assays

HEK293 transiently co-transfected with Bax and Flag-Bcl-x_L or Flag-Bcl-x_L deamidation mutants were used to assay the interaction with Bax 48 h post calcium-phosphate transfection. Cells were collected and were lysed in 1 × IP buffer (Sigma Aldrich). Co-immunoprecipitations were performed overnight with 1 mg of protein extracts and 2 µg of anti-Bax antibody (2D2, Santa Cruz Biotechnology). 50 µL of Protein G-agarose beads were added and incubated for 6 hours at 4°C. IPs were washed with 7 × 500 µL of 1 × IP buffer, and proteins were eluted with 30 µL of dissociation buffer (2% SDS, 125 mM Tris pH = 8.8, 20% glycerol, 0.002% (w/v) bromophenol blue).

Half of eluted fractions were separated on 12% SDS-PAGE prior to evaluation by western blots of the amount of immunoprecipitated Bax and co-immunoprecipitated Flag-Bcl-x_L.

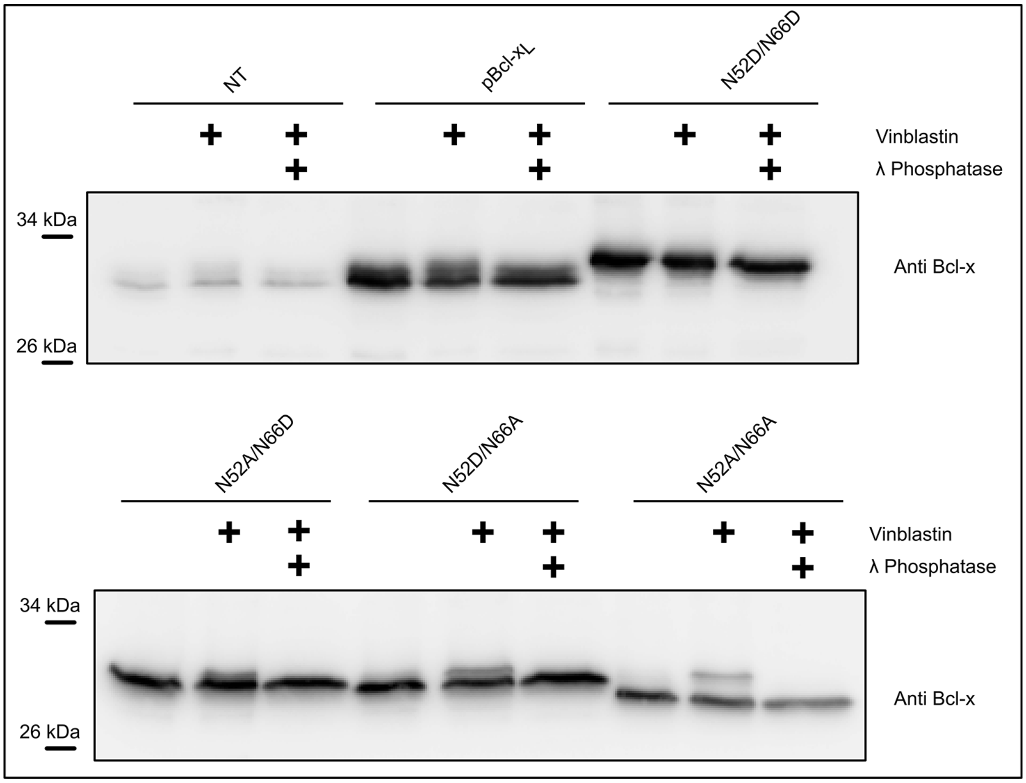

Supplementary Figure S1: HCT116 cells non transduced or transduced to express the indicated proteins were treated or not with 100 nM vinblastin for 24 h. Where indicated, samples treated with vinblastin were further incubated with lamba-phosphatase. Total proteins were extracted abd separated on SDS-PAGE. Immunodetection with anti-Bcl-x antibody was performed. Results are representative of 5 independent experiments.

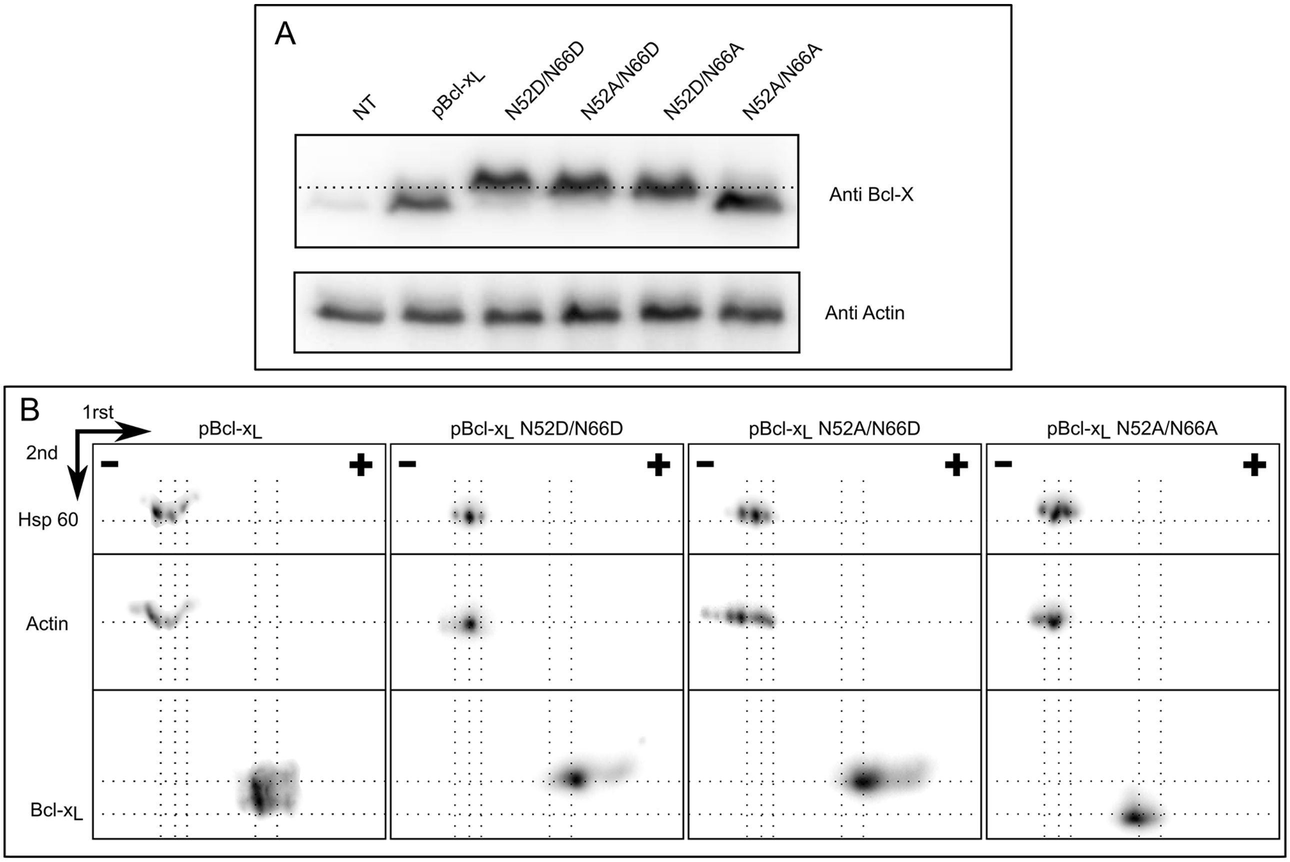

Supplementary Figure S2: (**A**) Stable HCT116 cells were generated by lentiviral transduction to overexpress either native Bcl-xL or the indicated deamidation mutants. Total proteins were extracted and 25 µg of proteins were analyzed on SDS-PAGE. (**B**) Total proteins extracted from the indicated cell lines were submitted to 2D electrophoresis: IEF in first dimension (polarity indicates migration toward the acidic side (+) and the basic side (–) of the IEF strip) and SDS-PAGE in second dimension. Western blots against Hsp60 and actin were used to establish the cartography of the migrations, and align the signals obtained after Bcl-x immunodetection. The single deamidation mutants exhibiting the same molecular weight and the same pI, only N52A/N66D is represented. Results are representative of at least 3 independent experiments.

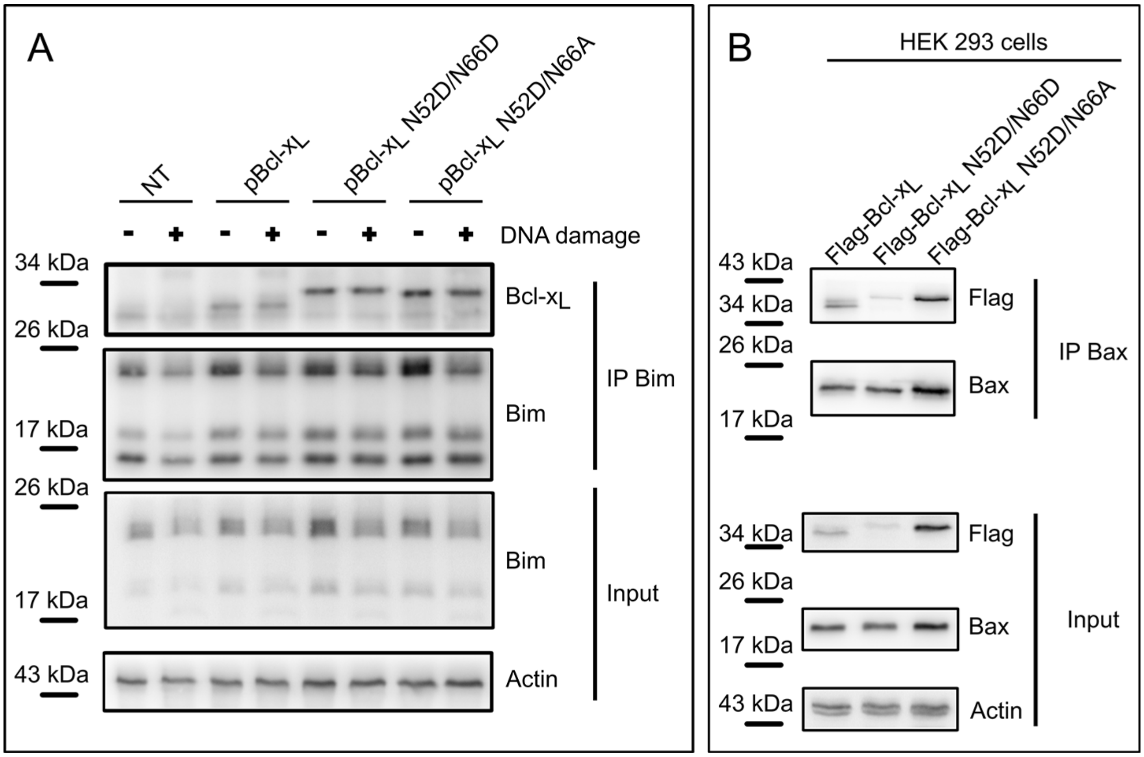

Supplementary Figure S3: (**A**) HCT116 cells non transduced or transduced to express the indicated proteins were submitted or not to DNA-damaging treatment (UV+5-FU). Immunoprecipitation was carried out with anti-Bim antibody, and revealed with anti-Bcl-x antibody. 30 µg of total cell extracts were separated on SDS-PAGE and immuno- detection against Bim and actin show the proteins levels in the input. Results are representative of at least 3 independent experiments. (**B**) HEK cells were transfected with the indicated constructs. Immuno¬precipitation was carried out with anti-Bax antibody, and revealed with anti-Bcl-x antibody. 30 µg of total cell extracts were separated on SDS-PAGE and immunodetection against Flag, Bax and actin show the proteins levels in the input. Results are representative of at least 3 independent experiments.

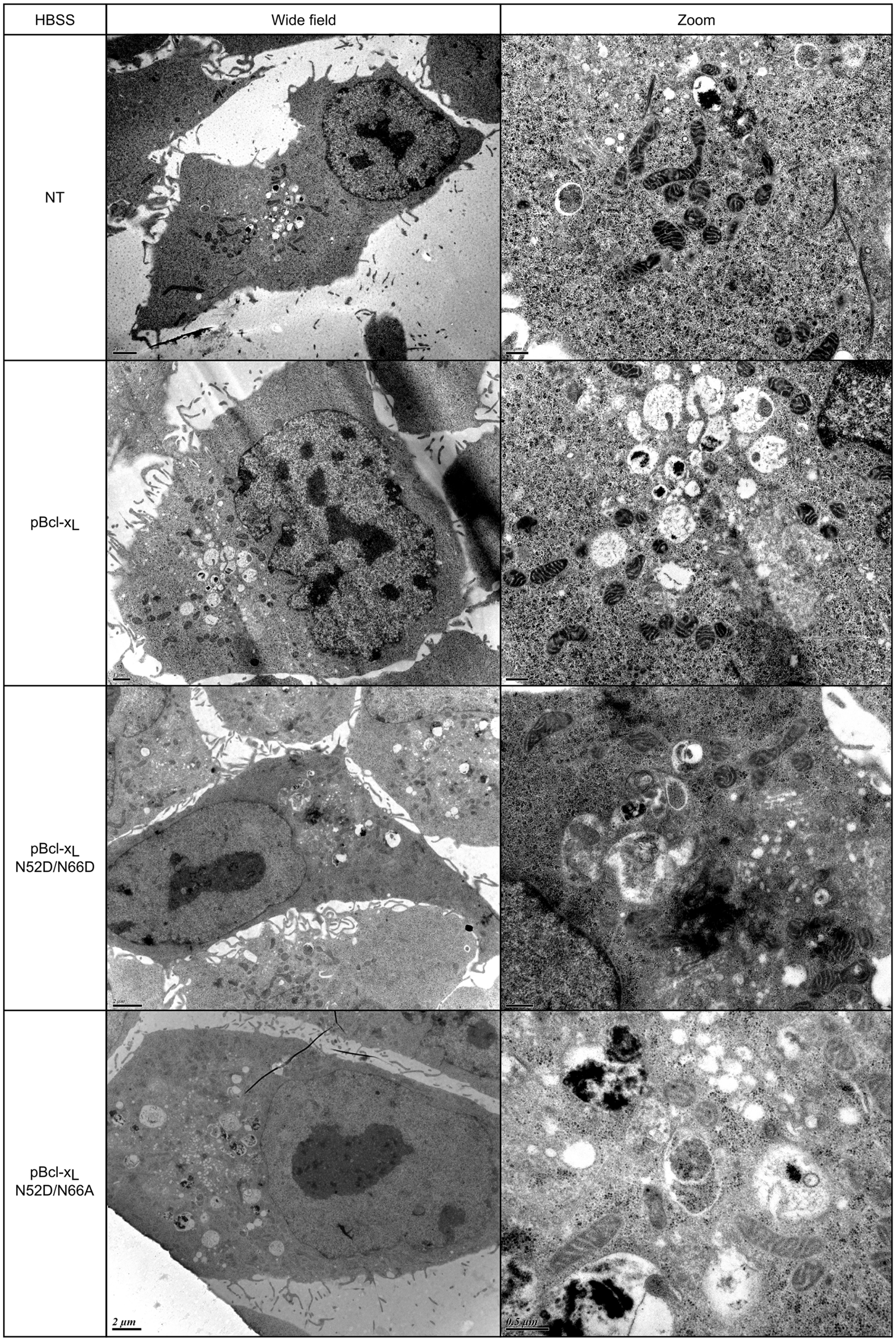

Supplementary Figure S4: HCT116 cells non transduced or transduced to express the indicated proteins were transferred for 6 h in HBSS supplemented with bafilomycin A1 to prevent degradation of autophagosomes. TEM were obtained as indicated in the Methods section. Wide sections are presented on the left, and a zoom of selected areas is shown on the right.
